# Implementation of baby boomer hepatitis C screening and linking to care in gastroenterology practices: a multi-center pilot study

**DOI:** 10.1186/s12876-016-0438-z

**Published:** 2016-04-04

**Authors:** Zobair M. Younossi, Louis L. LaLuna, John J. Santoro, Flavia Mendes, Victor Araya, Natarajan Ravendhran, Lisa Pedicone, Idania Lio, Fatema Nader, Sharon Hunt, Andrei Racila, Maria Stepanova

**Affiliations:** Center for Liver Diseases, Inova Fairfax Hospital, Falls Church, VA USA; Center for Outcomes Research in Liver Diseases, Washington, DC USA; Digestive Disease Associates, Wyomissing, PA USA; AGA Clinical Research Associates, LLC, Egg Harbor Township, NJ USA; GastroHealth, Miami, FL USA; Central Bucks Specialists, Gastroenterology, Doylestown, PA USA; Digestive Diseases Associates, Catonsville, MD USA; Cantara Clinical Solutions, LLC, Morristown, NJ USA; Betty and Guy Beatty Center for Integrated Research, Claude Moore Health Education and Research Building, 3300 Gallows Road, Falls Church, VA 22042 USA

## Abstract

**Background:**

Estimates suggest that only 20 % of HCV-infected patients have been identified and <10 % treated. However, baby boomers (1945-1965) are identified as having a higher prevalence of HCV which has led the Centers for Disease Control and Prevention to make screening recommendations. The aim of this study was to implement the CDC’s screening recommendations in the unique setting of gastroenterology practices in patients previously unscreened for HCV.

**Methods:**

After obtaining patient informed consent, demographics, clinical and health-related quality of life (HRQOL) data were collected. A blood sample was screened for HCV antibody (HCV AB) using the OraQuick HCV Rapid Antibody Test. HCV AB-positive patients were tested for presence of HCV RNA and, if HCV RNA positive, patients underwent treatment discussions.

**Results:**

We screened 2,000 individuals in 5 gastroenterology centers located close to large metropolitan areas on the East Coast (3 Northeast, 1 Mid-Atlantic and 1 Southeast). Of the screened population, 10 individuals (0.5 %) were HCV AB-positive. HCV RNA testing was performed in 90 % (9/10) of HCV AB-positive individuals. Of those, 44.4 % (4/9) were HCV RNA-positive, and all 4 (100 %) were linked to caregiver. Compared to HCV AB negative subjects, HCV AB-positive individuals tended to be black (20.0 vs. 5.2 %, *p* = 0.09) and reported significantly higher rates of depression: 60.0 vs. 21.5 %, *p* = 0.009. These individuals also reported a significantly lower HRQOL citing having more fatigue, poorer concentration, and a decreased level of energy (*p* < 0.05).

**Discussion:**

Although the prevalence of HCV AB-positive was low in previously unscreened subjects screened in the gastroenterology centers, the linkage to care was very high. The sample of patients used in this study may be biased, so further studies are needed to assess the effectiveness of the CDC screening recommendations.

**Conclusion:**

Implementation of the Baby Boomer Screening for HCV requires identifying screening environement with high prevalence of HCV+ individuals as well as an efficient process of linking them to care.

## Background

Hepatitis C viral (HCV) infection is the leading cause of cirrhosis and hepatocellular carcinoma in the United States, and the most common indication for liver transplantation [1–4]. There is increasing evidence that HCV is a systemic disease with both hepatic and extrahepatic manifestations [1]. There is also significant evidence that HCV infection is associated with tremendous economic burden including both direct and indirect costs associated with management of HCV-related hepatic and extrahepatic manifestations as well as lost years of life, impaired quality of life and work productivity [1–9] On the other hand, sustained viral response (SVR) of HCV infection has been reported to improve morbidity and mortality as well as health-related quality of life and work productivity in patients with HCV [10–13]. With the current all-oral second generation direct-acting antiviral agents, over 95 % of treated patients can achieve SVR with an excellent safety profile [14–29].

Despite substantial gains in treating HCV with these new highly effective antiviral regimens, there are a number of barriers which still exist [30–34]. Of these, the two most notable barriers are difficulty in obtaining insurance funding for the new regimens and the identification of all HCV infected patients [30–34]. The current estimates suggest that only between 10–50 % of HCV infected patients in the US are currently diagnosed [31]. This is partly due to health care providers’ lack of enthusiasm about the previous anti-HCV treatment regimens and their substantial side effect profile. Additionally, the recommended risk-based screening has not been effective in identifying infected patients [35]. Since 1998, the CDC has suggested HCV antibody screening of individuals with past behaviors or health indicators associated with HCV infection (e.g., history of injection drug use, hemodialysis, etc.). Despite these recommendations, more than 50 % of individuals with chronic hepatitis C (CHC) continue to be unaware of their infections, leading to questions about the effectiveness of such “risk-based” screening [36, 37].

In the United States, HCV infection is most prevalent among individuals born between 1945 and 1965 accounting for approximately 75 % of hepatitis C-associated mortality [38]. Since more than 50 % of infected individuals are unaware of their infection, the number of adults with CHC that will progress to cirrhosis, liver failure, hepatocellular carcinoma, and death is expected to increase dramatically in the coming decades [38, 39]. Without changes to CH-C screening, diagnosing and treatment paradigms, over the next 20 years, the total medical costs for individuals with HCV infection are expected to more than double, from $30 billion to over $85 billion [40]. Therefore, in 2012, CDC adjusted their recommendations to include a one-time hepatitis C screening of all individuals born between 1945 and 1965 [41]. The US Preventive Services Task Force has stepped forward and supported the CDC’s recommendation for birth cohort screening as well [42].

With therapies achieving SVR in >90 % of patients, targeted testing and link to care for infected persons in this birth cohort are expected to reduce HCV-related morbidity and mortality [35]. Therefore, the aim of this study was to implement a pilot screening project in 5 real world Gastroenterology Practices to identify baby boomers infected with HCV and to test the feasibility of screening and linking patients to care in a specialized practice setting.

## Methods

### Study population

This is a multi-center study sponsored by Chronic Liver Disease Foundation that involved 5 gastroenterology practices selected by the American College of Gastroenterology. The sites were large clinical practices within metropolitan areas that had familiarity with standard preventative screening procedures (e.g., colon cancer screening) and Good Clinical Practices (i.e., informed consent, data privacy, data collection). Enrollment started in 2014 and was completed in June 2015; it was competitive and not capped at any given site. Inclusion criteria were as follows: male and female patients born between 1945 and 1965, inclusive; willingness to give written informed consent; ability to read and understand English. Patients with documented history of HCV antibody (HCV Ab) or HCV RNA screening were excluded. To obtain baseline information, all patients were asked to fill out two questionnaires - one with their demographic and basic clinical history and one with health-related quality of life information (HRQL). Each patient underwent a blood draw to obtain a sample of blood for HCV screening. If the patient tested positive for HCV Ab, a standard of care confirmatory test was performed.

### Clinical and HRQL data

After informed consent, demographic, clinical and quality of life data were obtained. In particular, all enrolled individuals reported their age, gender, race/ethnicity and zip code. Medical history questionnaire asked about history of diabetes mellitus, hypertension, hyperlipidemia (that or high cholesterol or high triglycerides), heart disease (not specified), and about experiencing anxiety, depression, and fatigue. Individuals were also asked about recent alcohol consumption (3 or more drinks a week for a year) and about their current smoking status.

To assess fatigue, vitality, and exertion, 20 items from three widely used and extensively validated HRQL assessment instruments were selected and included into one questionnaire [43–47]. Specifically, the items were chosen from the physical functioning domain (PF) of the SF-36, the activity/energy (AE) and emotional (EM) domains of CLDQ-HCV, and the fatigue scale domain (FS) of FACIT-F [43–47]. The questionnaire was in English and self-administered. The responses to individual items were collected and, after transformation to a universal scale, were averaged to a total generic HRQL score (0–100).

### Screening for HCV infection

Sites were provided with commercially approved OraQuick HCV Rapid Antibody Test kits (OraSure Technologies). Each subject’s blood sample was screened for HCV Ab using Oraquick anti-HCV test. An adverse event was defined as any medical occurrence in response to the administration of the OraQuick Rapid HCV Test.

Individuals who were HCV Ab-negative were no longer followed-up for this study. For HCV Ab-positive individuals, a standard of care confirmatory testing was ordered by the screening site (as per standard medical practice) such as HCV RNA test, and results were collected. Individuals were also counseled and educated on HCV, including the use of alcohol, acetaminophen, and receiving hepatitis A and B vaccinations. The HCV Ab-positive individuals who consented to be followed also completed a four week follow-up HRQL survey. Finally, HCV RNA positive individuals were linked to care within the site practice or the geographical area and the date of the scheduled visit was recorded. The site also followed instructions regarding the local state requirements on whether a positive result had to be reported to a state health department.

### The study outcomes and statistical analysis

The primary endpoint of this study was the percentage of individuals with a positive HCV Ab. The secondary endpoints were the percentages of HCV Ab-positive individuals who underwent confirmatory testing and were linked to care, and HRQL scores at baseline and at follow-up.

The demographic and clinical parameters of individuals who were HCV Ab-positive or HCV Ab-negative were compared using Fisher exact test or Mann-Whitney non-parametric test. Individual HRQL items were treated as ordinal parameters; the total HRQL score was considered continuous. A p-value of less than 0.05 was considered significant. Independent predictors of a positive HCV Ab result were evaluated by a logistic regression using all collected clinico-demographic parameters as predictors. All analyses were run in SAS 9.3 (SAS Institute, Cary, NC). The study was approved by Copernicus IRB Board.

## Results

Two thousand baby boomer individuals were consented and screened in 5 gastroenterology practices selected by American College of Gastroenterology (regions: 3 Northeast, 1 Mid-Atlantic and 1 Southeast). The demographic and clinical data is summarized in Table 1, and HRQL data is summarized in Table 2.

**Table 1:** Demographics and medical history of the screened birth cohort

	HCV Ab+	HCV Ab-	p	All subjects
N	10	1,990		2,000
Age, years	58.4 ± 3.2	59.8 ± 6.0	0.41	59.8 ± 6.0
Race or ethnicity				
Caucasian	8 (80.0 %)	1429 (71.8 %)	0.73	1437 (71.9 %)
African-American	2 (20.0 %)	103 (5.2 %)	0.0933	105 (5.3 %)
Hispanic	0 (0.0 %)	418 (21.0 %)	0.13	418 (20.9 %)
Asian	0 (0.0 %)	26 (1.3 %)	1.00	26 (1.3 %)
Other	0 (0.0 %)	14 (0.7 %)	1.00	14 (0.7 %)
Male gender	4 (40.0 %)	793 (39.8 %)	1.00	797 (39.9 %)
History of:				
Type 2 diabetes	3 (30.0 %)	308 (15.5 %)	0.20	311 (15.6 %)
Hypertension	5 (50.0 %)	858 (43.4 %)	0.75	863 (43.4 %)
Hyperlipidemia	4 (40.0 %)	951 (47.9 %)	0.76	955 (47.8 %)
Anxiety	4 (40.0 %)	600 (30.3 %)	0.50	604 (30.3 %)
Depression	6 (60.0 %)	424 (21.5 %)	0.0094	430 (21.7 %)
Fatigue	6 (60.0 %)	742 (37.5 %)	0.19	748 (37.6 %)
Heart disease	2 (20.0 %)	219 (11.1 %)	0.31	221 (11.1 %)
Alcohol consumption > 30 g/week	3 (30.0 %)	545 (27.5 %)	1.00	548 (27.5 %)
Current smoking	3 (30.0 %)	199 (10.1 %)	0.0731	202 (10.2 %)

**Table 2:** Quality of life in the screened birth cohort

Question text	Range ^a^	Instrument, item (domain)	HCV Ab + (N = 10)	HCV Ab- (*N* = 1,889)	p	All subjects
How much have you been tired or fatigued during the last 2 weeks?	1–7	CLDQ-HCV Q1 (AE)	3.60 ± 1.96	4.82 ± 1.70	0.0412	4.82 ± 1.70
How much difficulty have you had with bending, lifting, or stooping in the last 2 weeks?	1–7	CLDQ-HCV Q4 (AE)	5.10 ± 1.85	5.53 ± 1.69	0.37	5.53 ± 1.69
How often during the last 2 weeks have you felt a decreased level of energy?	1–7	CLDQ-HCV Q7 (AE)	3.80 ± 2.10	4.98 ± 1.69	0.0155	4.97 ± 1.69
How often during the last 2 weeks have you felt depressed?	1–7	CLDQ-HCV Q16 (EM)	4.40 ± 2.12	5.88 ± 1.49	0.0144	5.87 ± 1.50
How much of the time during the last 2 weeks have you had problems concentrating?	1–7	CLDQ-HCV Q18 (AE)	4.80 ± 1.75	5.70 ± 1.47	0.0471	5.70 ± 1.47
*The following questions are about activities you might do during a typical day. Does your health now limit you in these activities? If so, how much?*						
Vigorous activities, such as running, lifting heavy objects, participating in strenuous sports	1–3	SF-36 PF01	2.10 ± 0.99	2.13 ± 0.82	0.41	2.13 ± 0.82
Moderate activities, such as moving a table, pushing a vacuum cleaner, bowling, or playing golf	1–3	SF-36 PF02	2.20 ± 1.03	2.60 ± 0.67	0.0122	2.59 ± 0.67
Lifting or carrying groceries	1–3	SF-36 PF03	2.30 ± 0.95	2.67 ± 0.60	0.0450	2.67 ± 0.60
Climbing several flights of stairs	1–3	SF-36 PF04	2.20 ± 0.92	2.47 ± 0.72	0.29	2.47 ± 0.72
Climbing one flight of stairs	1–3	SF-36 PF05	2.20 ± 1.03	2.69 ± 0.60	0.0064	2.68 ± 0.61
Bending, kneeling, or stooping	1–3	SF-36 PF06	2.40 ± 0.84	2.47 ± 0.70	0.65	2.47 ± 0.70
Walking more than a mile	1–3	SF-36 PF07	2.20 ± 0.79	2.46 ± 0.76	0.22	2.46 ± 0.76
Walking several hundred yards	1–3	SF-36 PF08	2.10 ± 0.88	2.66 ± 0.64	0.0195	2.66 ± 0.64
Walking one hundred yards	1–3	SF-36 PF09	2.10 ± 0.99	2.73 ± 0.58	0.0063	2.72 ± 0.59
Bathing or dressing yourself	1–3	SF-36 PF10	2.60 ± 0.84	2.86 ± 0.44	0.10	2.86 ± 0.44
*Please indicate how true each statement has been for you during the past 7 days.*						
I feel fatigued	4–0	FACIT-F HI7 (FS)	2.10 ± 1.45	1.11 ± 1.20	0.0107	1.11 ± 1.20
I feel tired	4–0	FACIT-F An2 (FS)	2.00 ± 1.49	1.25 ± 1.16	0.15	1.25 ± 1.16
I have trouble starting things because I am tired	4–0	FACIT-F An3 (FS)	1.70 ± 1.64	0.80 ± 1.12	0.0148	0.80 ± 1.13
I have energy	0–4	FACIT-F An5 (FS)	1.80 ± 0.63	2.41 ± 1.18	0.0352	2.41 ± 1.18
I need help doing my usual activities	4–0	FACIT-F An14 (FS)	0.80 ± 0.79	0.37 ± 0.86	0.0100	0.37 ± 0.86
Average score	0–100	generic	58.9 ± 32.5	76.3 ± 21.3	0.071	76.2 ± 21.4

^a^ the range is from the worst to the best health status. Higher scores indicate better HRQOL

Screened individuals were, on average, 60 ± 6 years old, 40 % male, 72 % Caucasian, 21 % Hispanic and 5 % African-American. Also, 30 % reported history of anxiety, 22 % reported depression and 38 % reported clinically overt fatigue. Furthermore, 16 % had a history of diabetes, 43 % had a history of hypertension, and 48 % had history of hyperlipidemia. Additionally, 27.5 % reported drinking alcohol ≥3 drinks per week, and 10.2 % reported current smoking (Table 1).

Of the screened population, 10 individuals (0.5 %) had positive serology for HCV. Of those, 4 (40 %) reported history of IV drug use and 2 (20 %) a history of intranasal drug use, 4 (40 %) had an unregulated tattoo, 1 (10 %) had a history of incarceration, and 1 (10 %) reported a history of blood transfusion before 1992 (Table 3).

**Table 3:** Additional socio-demographic information and link to care for HCV Ab + patients (*N* = 10)

	N (%) or mean ± std.dev.
Confirmed HCV ab+	10 (100.0 %)
HCV RNA-positive ^a^	4 (44.4 %)
ALT, IU/mL	32.8 ± 35.4
AST, IU/mL	25.2 ± 21.7
History of:	
Past or current IV drug use	4 (40.0 %)
Blood transfusions before 1992	1 (10.0 %)
Long-term hemodialysis	0 (0.0 %)
Incarceration	1 (10.0 %)
Being born to HCV-infected mother	0 (0.0 %)
Intranasal drug use	2 (20.0 %)
Unregulated tattoo or other percutaneous exposure	4 (40.0 %)
Counseled about:	
Acetaminophen use	9 (90.0 %)
Alcohol consumption	10 (100.0 %)
Hepatitis A and B vaccination	9 (90.0 %)
Linked to care	9 (90.0 %)
Completed follow-up questionnaire	6 (60 %)

^a^one HCV ab + patient refused to give blood sample

The HCV RNA testing was done in 90 % (9/10) of HCV-antibody positive individuals, and 44.4 % (4/9) were found to be HCV RNA-positive, 100 % of whom were counseled and linked to care by establishing an appointment regarding their HCV. Compared to HCV-antibody negative individuals, those who were HCV-antibody positive tended to be African-Americans (20.0 vs. 5.2 %, *p* = 0.09) and report more frequently a history of depression: 60.0 vs. 21.5 % (*p* = 0.009) (Table 1). Multivariate analysis with logistic regression showed that depression was the only clinical parameter independently associated with being HCV-antibody positive [odds ratio (95 % confidence interval) = 5.49 (1.54–19.54)].

The HCV-antibody positive individuals also had lower quality of life as documented by more fatigue, poorer concentration, less activity, and decreased levels of energy (all p-values < 0.05) (Table 2). The four weeks follow-up HRQOL questionnaire showed no significant changes (all *p* > 0.1) from the baseline values in individuals who tested positive for HCV (Table 4).

**Table 4:** Follow-up HRQL questionnaire in HCV Ab + patients (*N* = 6)

Question text	Range ^a^	Instrument, item (domain)	baseline	4 week f/u
How much have you been tired or fatigued during the last 2 weeks?	1–7	CLDQ-HCV, Q1 (AE)	4.00 ± 1.26	4.50 ± 1.64
How much difficulty have you had with bending, lifting, or stooping in the last 2 weeks?	1–7	CLDQ-HCV, Q4 (AE)	5.17 ± 1.83	5.33 ± 1.37
How often during the last 2 weeks have you felt a decreased level of energy?	1–7	CLDQ-HCV, Q7 (AE)	4.00 ± 1.55	4.50 ± 1.64
How often during the last 2 weeks have you felt depressed?	1–7	CLDQ-HCV, Q16 (EM)	5.17 ± 1.94	5.00 ± 1.55
How much of the time during the last 2 weeks have you had problems concentrating?	1–7	CLDQ-HCV, Q18 (AE)	5.17 ± 1.17	5.50 ± 1.05
*The following questions are about activities you might do during a typical day. Does your health now limit you in these activities? If so, how much?*				
Vigorous activities, such as running, lifting heavy objects, participating in strenuous sports	1–3	SF-36, PF01 (PF)	2.17 ± 0.98	2.00 ± 0.89
Moderate activities, such as moving a table, pushing a vacuum cleaner, bowling, or playing golf	1–3	SF-36, PF02 (PF)	2.33 ± 1.03	2.33 ± 0.82
Lifting or carrying groceries	1–3	SF-36, PF03 (PF)	2.50 ± 0.84	2.33 ± 0.82
Climbing several flights of stairs	1–3	SF-36, PF04 (PF)	2.33 ± 0.82	2.17 ± 0.98
Climbing one flight of stairs	1–3	SF-36, PF05 (PF)	2.33 ± 1.03	2.17 ± 0.98
Bending, kneeling, or stooping	1–3	SF-36, PF06 (PF)	2.50 ± 0.84	2.00 ± 0.89
Walking more than a mile	1–3	SF-36, PF07 (PF)	2.17 ± 0.75	2.17 ± 0.98
Walking several hundred yards	1–3	SF-36, PF08 (PF)	2.00 ± 0.89	2.33 ± 0.82
Walking one hundred yards	1–3	SF-36, PF09 (PF)	2.00 ± 1.10	2.33 ± 0.82
Bathing or dressing yourself	1–3	SF-36, PF10 (PF)	2.67 ± 0.82	2.67 ± 0.82
*Please indicate how true each statement has been for you during the past 7 days.*				
I feel fatigued	4–0	FACIT-F HI7 (FS)	2.00 ± 1.10	1.33 ± 1.51
I feel tired	4–0	FACIT-F An2 (FS)	2.00 ± 1.41	1.50 ± 1.38
I have trouble starting things because I am tired	4–0	FACIT-F An3 (FS)	1.33 ± 1.37	1.33 ± 1.21
I have energy	0–4	FACIT-F An5 (FS)	2.00 ± 0.63	1.67 ± 0.52
I need help doing my usual activities	4–0	FACIT-F An14 (FS)	0.50 ± 0.55	1.00 ± 0.89
Average score	0–100	Generic	63.1 ± 29.9	63.4 ± 27.9

^a^the range is from the worst to the best health status

## Discussion

This is the largest HCV screening program in the baby boomers who presented to a specialty gastroenterology practices for clinical care. The data suggest that the prevalence of HCV antibody positivity in this particular study setting (subjects visiting GE practices who have not previously been identified) is relatively low (0.5 %) as compared to the reported HCV AB positive rate in the general population at 4.1 % for males and 1.6 % for females [48]. It is important to note that, in addition to a bias introduced solely by the fact of patients being seen in tertiary care centers in a small sample of localities, our study excluded individuals who had already been diagnosed with HCV or had been screened for HCV previously due to meeting certain high risk criteria. Therefore, the prevalence rate reported in this study is likely substantially lower than one that could have been obtained in a community-based screening setting, and neither does it reflect the true HCV prevalence in the GE practices (due to exclusion of those with an existing diagnosis of HCV).

Our study also indicates that about half of the individuals who were HCV antibody positive were viremic. In this HCV cohort, risk factors reported were similar to those previously known for HCV viremic patients [1–4]. Furthermore, African Americans tended to have a higher prevalence of HCV which is also consistent with previous reports. Additionally, those testing HCV positive had more depression independently associated with their HCV positivity status. All HCV positive individuals were linked to follow-up care through scheduled appointments. This is a significant finding as this part of the screening, diagnosis, and treatment continuum has been a challenge in other settings such as emergency rooms [2].

These data are similar to those reported by Sears et al. from a single GE practice for baby boomers undergoing colonoscopy. In fact, in that study, only one of 376 subjects (0.27 %) was HCV RNA positive, a rate almost identical to our HCV viremic individuals (0.2 %) [3]. There are a number of potential explanations for the relatively low prevalence of HCV in GE practices. The most important explanation may be related to the type of patients who are seen in GE practices – they are most likely insured with the majority (72 %) being Caucasians.

The low prevalence of HCV in this special population as compared to a community-based approach is of special importance as specialty practices may not be the best places to screen for HCV but may provide the best avenue for follow-up care once identified. For instance, Galbraith et al. reported screening of baby boomers in an emergency room. These authors reported an 11.1 % positive HCV antibody rate with 68 % being viremic. On the other hand, only 54 % of HCV viremic patients were able to be contacted and 38 % were able to be scheduled for follow-up appointments indicating a significant drop-off [2].

In addition to high prevalence of HCV in the baby boomers seen in the emergency department, the prevalence rates are also high for baby boomers screened in the hospital setting. In a study by Turner et al., the prevalence of newly diagnosed HCV in hospitalized baby boomers was 8 % [4]. Finally, in a study reported by Morano et al., baby boomers were screened either by point of care (POC) HCV antibody testing or traditional serologic testing in the setting of a mobile medical clinic. The reported prevalence of HCV positivity in this cohort was 6.2 %. Individuals who underwent POC testing were much more likely (93.8 %) than traditional serologic testing (18.2 %) to be linked to care [5]. In a recent modeling study from 15 countries worldwide, investigators found that diagnosing and treating a small proportion of patients with high efficacy drugs can have a significant effect on the reduction of the HCV disease burden within the countries studied [49]. At the same time, the authors caution that the best scenario would be to have increased diagnosis and treatment with high efficacy treatments to have the best results. Others, though, argue that the model used was not a dynamic model and thus may not capture any new infection or reinfections so may overestimate the true impact of the use of high efficacy drugs [50].

These findings not only assist healthcare workers in identifying better areas for the identification of HCV but may assist healthcare workers in providing a better method to link screening with follow-up care. Link to care is important to deliver the highly effective antiviral treatment to patients with HCV. Our data suggest high rates of linking to care for patients who are HCV-positive to a GE clinic. Nevertheless, given the small sample size, the generalizability of this data must be interpreted with caution. Furthermore, if a GE clinic is not available another suggested method is the use of Innovative Mobile Clinics equipped with POC testing for HCV that use established pathways [5]. Using mobile clinics allow for HCV-positive patients to be immediately linked to care.

In this study, we also assessed the quality of life in all patients at baseline before they knew their HCV infection status as there are previous data which suggest that knowledge about HCV diagnosis can impair HRQL [51]. We have found that at baseline patients who were HCV AB+ had more impairment of their HRQL having more complaints of fatigue, poorer concentration, less activity, and decreased levels of energy as compared to those who were HCV AB-. This is consistent with previous data which have demonstrated that HCV-infected patients suffer from HRQL impairment possibly due to the potential effects of the virus crossing the blood-brain barrier and affecting the brain chemistry directly [52–54]. In our study, for the patients who were HCV AB+, a follow-up survey was administered 4 weeks after their initial diagnosis, and there were no statistically significant change in their reported HRQL indicating that knowing the status of being HCV AB+ did not influence their HRQL. However, the small sample size of our study may have contributed to our inability to detect this difference in the HRQOL scores.

One of the limitation of this study was the study population referred to a GE practice indicating access to insurance coverage for consultative services and colonoscopy. This could potentially introduce a bias by excluding uninsured individuals who are known to have high prevalence of HCV (30). Another limitation of our study was our focus on “the age cohort” as the risk factor for HCV. Although this was done to determine the prevalence of HCV solely based on the age-based risk factor, other risks were not included.

## Conclusions

In summary, our data show that the outcome of screening and then linkage to care for the baby boomers found to be HCV-positive is feasible but may depend on the setting. In this study, GE practices appeared to have a low prevalence of HCV, but the linkage to care occurred universally. Therefore, a strategy to maximize both the yield of HCV screening and linkage to care with appropriate providers will be critical for identifying and successfully treating patients infected with HCV.
